# 

**Published:** 1867-07

**Authors:** 


Vol. VIII. JULY, 1867. No. 5.

### ATLANTA Medical and Surgical JOURNAL.

NEW SERIES.

EDITED BY

J. G. WESTMORELAND, M. D.,
*Professor of Materia Medica and Therapeutics in the Atlanta Medical College.*

W. F. WESTMORELAND, M. D.,
*Professor of the Principles and Practice of Surgery in the Atlanta Medical College.*

AND

J. M. JOHNSON, M. D.

**Pax et scientia, sed veritas sine timore.**

**PUBLISHED MONTHLY AT $4.00 PER ANNUM, IN ADVANCE**

ATLANTA, GA.:
PRINTED AT THE ATLANTA INTELLIGENCER BOOK & JOB OFFICE.
1867.

Postage 36 cts. a year, pre-paid quarterly. Postmasters are requested to compl with the law, bv giving us notice when the Journal is refused at their Offices.

